# Data envelopment analysis on the efficiency of vaccination services and its influencing factors in Beijing, China

**DOI:** 10.1186/s12913-023-09758-0

**Published:** 2023-07-08

**Authors:** Mingzheng Hu, Yanshang Wang, Ming Wang, Dawei Zhu, Wentao Li, Rui Yu, Jiang Wu, Min Lv, Ping He

**Affiliations:** 1 School of Public Health, Peking University, 38 Xue Yuan Road, Haidian District, Beijing, 100191 China; 2 China Center for Health Development Studies, Peking University, 38 Xue Yuan Road, Haidian District, Beijing, 100191 China; 3 Institute for Immunization and Prevention, Beijing Center for Disease Prevention and Control, Beijing, China

**Keywords:** Vaccination, Efficiency, DEA, Influencing factors, China

## Abstract

**Objectives:**

Vaccination is an important part of public health services. We aim to assess the efficiency of vaccination services in Beijing, the capital of China, and to further study the influencing factors of efficiency.

**Methods:**

Using the immunization service data of Beijing, China in 2020, we firstly developed a data envelopment analysis (DEA) model to calculate the score of vaccination efficiency. Secondly, we used DEA model scenario simulations with different combinations of input–output factors to derive the magnitude of the effect of each input factor on the efficiency. Finally, combined with the data from the Beijing Regional Statistical Yearbook 2021, we developed the Tobit model to examine the effect of external social environmental factors on efficiency.

**Results:**

The average scores of efficiency of POVs (Point of Vaccination) in different areas of Beijing vary greatly. Different input factors had different degrees of positive effects on the efficiency score. In addition, the number of populations served by POV was positively associated with efficiency, the GDP and financial allocation of the POVs’ district was also positively associated with efficiency score, while the total dependency ratio of the POVs’ district was negatively associated with efficiency score.

**Conclusion:**

The efficiency of vaccination services varied considerably across POVs. Constrained by limited resources, efficiency scores can be increased by increasing input factors that have a larger impact on efficiency score and reducing those that have a smaller impact on efficiency. In addition, the social environment should be considered in allocating vaccination resources, and more resources should be invested in areas with low levels of economic development, low financial allocation, and high population.

## Introduction

Vaccination is an economical and effective means of preventing and controlling infectious diseases [1]. To improve the immunity of the world's population, the Expanded Program on Immunization (EPI) was introduced by the World Health Organization (WHO) in the 1970s, which led to the introduction of the Chinese national immunization program in 1978. Since then, to increase vaccine coverage, many scholars have studied the factors that influence immunization rates [2–6], and with the joint efforts of scholars and the government, China has achieved over 90% immunization program coverage.

However, the high immunization program coverage was accompanied by countless financial investments. According to China's National Bureau of Statistics, the Chinese government needs to invest about RMB 4 billion annually to purchase vaccines and medical instruments such as syringes, and the amount is becoming larger as the coronavirus vaccine has begun its mass vaccination since COVID-19. Faced with such a huge amount of financial expenditure, we cannot help but start thinking: Are these huge financial health resources being used efficiently? What are the factors that affect this efficiency? How to improve efficiency to reduce the cost? This series of questions are worthy of our in-depth consideration.

Although the research on efficiency assessment in health care is growing [7–9], there is a very limited number of studies regarding the efficiency of immunization programs [10–12], which makes these questions cannot be answered. Among the limited studies, Hollingsworth et al. [10] calculated the efficiency of child immunization programs in Australia using the DEA method and found that urban areas were more efficient than rural areas [10]. Besides, Menzies et al., [11] calculated the efficiency of childhood vaccination in six countries using the DEA method and the SFA method separately and found that within each country, efficiency varied widely and that the result of SFA was systematically higher than DEA [11]. Moreover, Lobo et al. [12] used the DEA method to evaluate the efficiency of the Brazilian National Immunization Program (NIP) [12]. However, these studies have certain limitations. First, they calculated vaccination efficiency and conducted a descriptive analysis of efficiency, but did not explore which factors will influence the efficiency and to what extent these factors will influence efficiency. Second, when calculating the vaccination efficiency, they only included the number of children's vaccines, which ignored the number of adult vaccines. However, POV serves both children and adults, while adult immunization is also an important public health issue. Therefore, it is necessary to include the amount of adult vaccination in the calculation.

In this study, we attempted to explore the factors influencing vaccination efficiency and give policy recommendations to improve vaccination efficiency. Beijing's immunization program provides free and self-funded vaccines for children and adults, and the vaccination volume of children and adults is very large, which provides a good sample for the study of this problem. Hence, using the immunization service data of Beijing, China in 2020, we first got the score of vaccination efficiency in Beijing by employing the DEA model, to assess the efficiency of vaccination in Beijing. Secondly, to derive the magnitude of the effect of each input factor on the efficiency, we conducted DEA model scenario simulations with different combinations of input–output factors. Thirdly, since the output of the social sector does not only depend on these input variables but is also influenced by the external social environment [13], such as macroeconomics and population size, we further used the Tobit model to investigate the effect of external social environmental factors on efficiency.

Our research has the following contributions: Firstly, to our knowledge, this is the first study to examine the factors influencing the efficiency of vaccination services in China, which not only enriches the research on the factors influencing vaccination efficiency but also provides empirical lessons for other developing countries with similar backgrounds to improve vaccination efficiency. Secondly, unlike previous studies that mainly focused on the efficiency of child vaccination [10–12], our research results measure the overall efficiency of child and adult vaccination, providing a more comprehensive reference for the government to formulate vaccination policies.

## Methods

### Data source

In terms of variables of the DEA model, the Beijing Center for Disease Prevention and Control (CDC) provided the data of 454 POVs in the immunization program in Beijing in 2020. After removing data with missing values, 418 POVs remained. In addition, the data for the Tobit model came from the *Beijing Regional Statistical Yearbook 2021* and Beijing CDC. Data at the district area level were matched to clinical data based on the geographic location of the POVs.

### Measures

#### Input and output variables

In terms of input indicators, by referring to the Cobb–Douglas Production Function [14] shown in Eq. (1), this paper developed input indicators from the three dimensions: capital (K), labor (L), and technology (T). In addition, in terms of output indicators (Y), referring to other scholars' studies [11], we used the amounts of vaccination.

1 $$Y=F(K,L,T)$$

Considering the availability of data, we selected input–output indicators from these dimensions as shown in Table 1. Firstly, the higher the level of the POVs, the more capital investment is required to purchase infrastructure, train staff, etc. Therefore, the level of the POVs can represent the capital input. Second, having more staff means having more people in the workforce to invest. More days open for children and adults, working on weekends, means more labor intensity invested. Thus, these variables can represent labor input. Thirdly, a higher proportion of staff with a bachelor's degree indicates the medical technology of the clinic staff is higher. Moreover, having an information technology management logo reflects a higher level of investment in modern electronic technology. Therefore, these two variables can be used to represent the level of technological input. Fourth, as for output variables, since different vaccination types may have different influences on efficiency, we divided the amount of vaccination into four types to facilitate the exploration of the separate effects of different vaccination amounts on efficiency.

**Table 1:** Input–output indicators of vaccination services

Type	Indicator Name	Symbol
Capital input	POVs level	${X}_{1}$
**L**abor input	Number of staff	${X}_{2}$
**L**abor input	Number of days open for children	${X}_{3}$
**L**abor input	Number of days open for adults	${X}_{4}$
**L**abor input	Whether open on weekends	${X}_{5}$
Technology input	Percentage of staff with bachelor's degree	${X}_{6}$
Technology input	Whether have information management logo	${X}_{7}$
Output	Amount of self-funded vaccination for kids	${Y}_{1}$
Output	Amount of free vaccination for kids	${Y}_{2}$
Output	Amount of self-funded vaccination for adults	${Y}_{3}$
Output	Amount of free vaccination for adults	${Y}_{4}$

Specifically, the POVs Level variable is assigned a value of 1–3 according to the POVs level, with higher values representing higher POVs levels; the Number of Staff variable is assigned a value based on the actual number of staff in each POV; the Number of Days Open for Children and the Number of Days Open for Adults variables are the actual number of days children or adults are open for vaccination in each POV; the Whether Open on Weekends variable is a dummy variable and is assigned a value of 1 if a POV is open on weekends and 0 if not; the Percentage of Staff with a Bachelor's Degree variable is the actual percentage of staff with a bachelor's degree or higher in a POV; the Whether Have Information Management Logo variable is a dummy variable, and is assigned a value of 1 if a POV has information management logo and 0 if not; the Amount of Self-funded or Free Vaccination for Kids or Adults variables are the actual number of each type of vaccinations given at each POV.

#### Tobit regression variables

The dependent variable was the efficiency scores calculated from the DEA model. Referring to related literature [15], the explanatory variables were developed from four dimensions: economic development indicators (logarithm of GDP per capita in 2020 at the district level), financial allocation indicators (logarithm of public finance spending in 2020 at the district level), environmental indicators (the annual average value of PM2.5 in 2020 at the district level), and demographic indicators (logarithm of the number of people served per POV in 2020, total dependency ratio in 2020 at the district level).

### Data envelopment analysis

#### DEA model development

Data Envelopment Analysis (DEA) is a method for evaluating the relative effectiveness of decision-making units (DMUs) with multiple inputs and outputs using mathematical programming [10]. Assumed that there are M immunization POVs whose input–output efficiency of immunization services needs to be assessed, and the system of indicators is assumed to be A input indicators and B output indicators. Assumed that ${X}_{ma}$ (${X}_{ma}>0$) represents the value of the ${A}_{th}$ input indicator for the ${M}_{th}$ immunization POV and ${Y}_{mb}$ (${Y}_{mb}>0$) represents the ${l}_{th}$ value for the ${M}_{th}$ immunization POV. For the m (m = 1, 2, …, M) immunization POV, $\theta \left(0\le \theta \le 1\right)$ represents the efficiency score; $\varepsilon$ is a non-Archimedean infinitesimal; ${\lambda }_{m} \left({\lambda }_{m}\ge 0\right)$ is a weighting variable to determine the size gain of the immunization POV; ${s}^{-} \left({s}^{-}\ge 0\right)$ is a slack variable that indicates the amount of input reduction needed for the immunization POV to reach DEA effectiveness; ${s}^{+} \left({s}^{+}\ge 0\right)$ is a residual variable that indicates the amount of output increase needed for the immunization POV to reach DEA effectiveness. The following Eq. 2 is the DEA model for measuring the input–output efficiency of immunization POVs. MAX DEA software was used to calculate the efficiency of the vaccination service.

2 $$\left\{\begin{array}{c}min\left[\theta -\varepsilon \left(\sum_{a=1}^{A}{s}^{-}+\sum_{b=1}^{B}{s}^{+}\right)\right]\\ s.t.\sum_{m=1}^{M}{x}_{ma}{\lambda }_{m}+{s}^{-}=\theta {x}_{a}^{m},a=\mathrm{1,2},3\dots ..A\\ \sum_{m=1}^{M}{y}_{mb}{\lambda }_{m}-{s}^{+}={y}_{b}^{m},b=\mathrm{1,2},3\dots ..B\\ \sum_{m=1}^{M}{\lambda }_{m}=1,m=\mathrm{1,2},3\dots ..M\end{array}\right.$$

#### SBM DEA model

To improve the robustness of the DEA model in calculating efficiency scores, the SBM DEA model has been further developed. The SBM DEA model is based on the assumption that inputs or outputs vary proportionally and does not take into account the slack in the indicators, whereas in the SBM DEA model the inputs and outputs do not need to vary strictly proportionally, which gives a more realistic measure of the efficiency of each decision unit, and therefore the results are more robust. The equation of the SBM DEA model is shown in [3], where $\rho$ is the efficiency value, ${s}^{-}$ and ${s}^{+}$ are the slack variables for inputs and outputs respectively, and $\lambda$ is the weight variable. ${x}_{a0}$, ${y}_{b0}$ are the a-th input and b-th output of the decision unit, respectively.

3 $$\left\{\begin{array}{c}min\rho \frac{1-\frac{1}{A}\sum_{a=1}^{A}\frac{{s}_{a}^{-}}{{x}_{a0}}}{1+\frac{1}{B}\sum_{b=1}^{B}\frac{{s}_{b}^{+}}{{y}_{b0}}}\\ s.t.{x}_{0}=X\lambda +{s}^{-}\\ {y}_{0}=Y\lambda -{s}^{+}\\ \lambda \ge 0,{s}^{-}\ge 0,{s}^{+}\ge 0\end{array}\right.$$

#### DEA model scenario simulation

To measure the extent to which each input indicator affects efficiency, scenarios can be conducted using different combinations of input–output indicators. Scenario simulation analysis was carried out using a combination of scenarios with one indicator removed in turn.

Assumed that $D$ is the set of input–output indicators, $V\left(D\right)$ is the mean score of DEA efficiency in the case of the initial indicator set $D$, ${D}_{i}$ is the set of indicators after removing the ${i}_{th}$ input indicator, $i\in \left[\mathrm{1,7}\right]$, and $V\left({D}_{i}\right)$ is the mean score of efficiency for each scenario. The degree of effect of each input indicator on the DEA efficiency can be calculated by the following Eq. (4). ${S}_{i}$ is the degree of affection of the ${i}_{th}$ input indicator on DEA efficiency, the larger ${S}_{i}$, the greater effect on DEA efficiency.

4 $${S}_{i}=\frac{V\left(D\right)-V({D}_{i})}{V({D}_{i})}\times 100\%$$

## Results

### Descriptive statistical analysis

Table 2 shows the descriptive statistical analysis of the variables in the DEA model and the Tobit model.

**Table 2:** Descriptive statistical analysis

Types	Variable	Mean	Std. Dev
Input	POVs level	1.624	0.696
	Number of staff	9.522	6.083
	Number of days open for children	1.931	1.283
	Number of days open for adults	1.748	1.396
	Whether open on weekends	0.043	0.203
	Percentage of staff with bachelor's degree	0.560	0.249
	Whether have information management logo	0.622	0.485
Output	Amount of self-funded vaccination for kids	11461.27	11171.24
	Amount of free vaccination for kids	4354.124	4670.556
	Amount of self-funded vaccination for adults	3082.519	2348.776
	Amount of free vaccination for adults	1969.598	2397.058
External	Logarithm of GDP per capita	10.989	0.312
	Logarithm of public finance spending	5.663	0.543
	Logarithm of the number of people served	9.944	1.778
	Total dependency ratio	0.345	0.056
	Annual average value of PM2.5	35.328	3.017

### Efficiency scores of vaccination services

Due to the sample size, it was too long to enumerate the efficiency values for each clinic by listing, so we categorized the POVs according to the districts they belonged to and calculated the average efficiency for each district (four decimal places were retained) in Table 3. The average vaccination efficiency in Beijing was 0.600 (DEA) and 0.4314 (SBM DEA), eight counties had a regional average efficiency higher than the city average, eight counties had a regional average efficiency lower than the city average, the highest average efficiency county having an efficiency value of 0.7989 (DEA) and 0.6943 (SBM DEA), and the lowest average efficiency county having an efficiency value of 0.3813 (DEA) and 0.1264(SBM DEA).

**Table 3:** Average efficiency scores for each district in Beijing

District	DEA	SBM DEA	District	DEA	SBM DEA
District 1	0.5382	0.3364	District 9	0.6882	0.5008
District 2	0.5690	0.4254	District 10	0.5429	0.3227
District 3	0.7699	0.6635	District 11	0.6507	0.4944
District 4	0.6140	0.4845	District 12	0.7443	0.5469
District 5	0.4933	0.3567	District 13	0.4857	0.2367
District 6	0.6287	0.5045	District 14	0.7989	0.6943
District 7	0.3813	0.2813	District 15	0.4710	0.1264
District 8	0.4829	0.2337	District 16	0.3940	0.2010

**Fig. 1:**
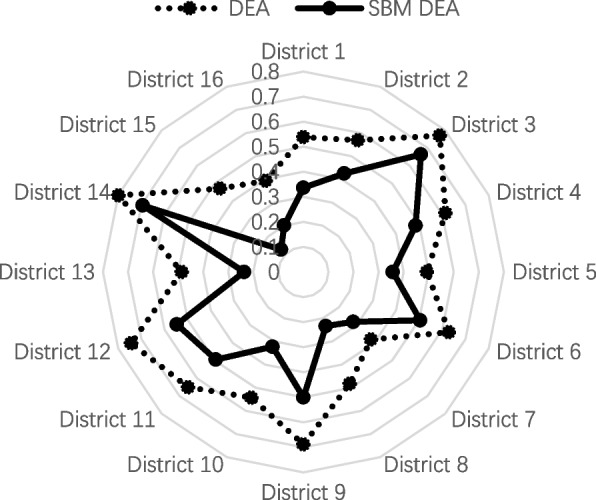
The radar chart of average efficiency scores for each district in Beijing

Additionally, to improve the clarity and readability, we further provide a radar chart of the efficiency scores in Fig. 1, as can be seen in Fig. 1, the results of the DEA model are considerably biased against the results of the SBM DEA model, indicating that the SBM DEA model is more meaningful and robust for estimation. Therefore, the SBM DEA model results were used for the efficiency values in the next DEA scenario simulations as well as in the Tobit model regression.

### DEA model scenario simulation

Table 4 shows the results of the scenario simulation. The labor, capital, and technology factors all contribute positively to efficiency, with the effecting degree of 3.71% on the POV level, 15.99% on the number of staff, 7.47% on the number of days open for kids, 7.06% on the number of days open for adults, 1.36% on whether open on weekends, 10.24% on the percentage of staff with bachelor's degree, and 13.76% on whether have information management logo.

**Table 4:** Scenario simulation results

No	Indicators removed	Indicators remained	$\overline{{\varvec{\theta}} }$	Effecting degree
1	POVs level	${X}_{2-7}$、${Y}_{1-4}$	0.4154	3.71%
2	Number of staff	${X}_{1}$、${X}_{3-7}$、${Y}_{1-4}$	0.3624	15.99%
3	Number of days open for kids	${X}_{1-2}$、${X}_{4-7}$、${Y}_{1-4}$	0.3992	7.47%
4	Number of days open for adults	${X}_{1-3}$、${X}_{5-7}$、${Y}_{1-4}$	0.4010	7.06%
5	Whether open on weekends	${X}_{1-4}$、${X}_{6-7}$、${Y}_{1-4}$	0.4256	1.36%
6	Percentage of staff with bachelor's degree	${X}_{1-5}$、${X}_{7}$、${Y}_{1-4}$	0.3873	10.24%
7	Whether have information management logo	${X}_{1-6}$、${Y}_{1-4}$	0.3721	13.76%

### Tobit model results

As shown in Table 5, there were four significant variables in the regression results. The indicators of economic development and financial allocation were significant at the 1% level with a coefficient of 0.376 and 0.176, respectively. Besides, the logarithm of the number of people served and total dependency ratio were also significant with a regression coefficient of 0.085 and -1.157, respectively. However, PM2.5 was not significant with a regression coefficient of -0.012. Additionally, as reported in Table 5, an OLS linear regression model was applied to check the robustness of the results. The significance of the regression coefficients is consistent with the results of the Tobit regression model, which verify the robustness of the results.

**Table 5:** Regression results

	Vaccine efficiency	
	Tobit	OLS
GDP	0.376 ^***^	0.310^***^
	(0.111)	(0.093)
Financial Allocation	0.176^***^	0.142^***^
	(0.065)	(0.055)
Population Served	0.085^***^	0.071^***^
	(0.013)	(0.011)
Dependency Ratio	-1.157^**^	-0.990^**^
	(0.516)	(0.436)
PM2.5	-0.012	-0.006
	(0.012)	(0.010)
Constant Term	-4.768^***^	-4.002^***^
	(0.936)	(0.781)
${R}^{2}$	0.1965	0.2204

^***^ *p* < 0.01, ** *p* < 0.05

## Discussion

Improving the efficiency of vaccine services is important for reducing government spending on health and increasing full vaccine coverage. From this point of view, DEA is a good analytical tool for exploring efficiency issues. In this study, we first measured the efficiency of 418 vaccination POVs in Beijing in 2020 using the DEA method. Secondly, to investigate whether there is an impact of different input and output factors on efficiency and which factor has a greater degree of impact on efficiency, we conducted scenario simulations for different input–output combinations. Finally, since healthcare is a complex system whose efficiency is also influenced by the external social environment, such as geography, socio-economics, and demographics [16], we further developed the Tobit model to analyze the impact of the external social environment factors on efficiency.

Our result indicated that the mean efficiency of immunization in Beijing is 0.6000 (DEA) and 0.4314 (SBM DEA). Compared with the previous research, the efficiency is higher than that of developing countries [11] in Africa, Latin America, and Europe, but relatively lower than that of developed countries such as Australia [10]. The reason for our lower results may be that the previous studies only calculated the number of vaccinations for children, whereas our studies included the number of vaccinations for adults and children. However, the number of vaccinations for adults is much smaller than that for children, so our average efficiency is relatively low. In addition, we also found the efficiency varied considerably between different POVs in Beijing, which was consistent with the prior studies [10, 11].

The result of scenario simulations showed that all the input factors of the POV had a positive impact on efficiency. First, in terms of capital inputs, the grade of the POV contributed positively to the efficiency. This may be because a higher POV grade means more capital investment, so the POV will have more money to purchase advanced equipment and hire highly educated staff, resulting in higher efficiency. This is similar to the findings on hospital efficiency from other countries, such as Serbia [17], Palestine [18], and Turkey [19]. Second, labor factor inputs, including the number of staff, the number of opening days, and whether opening on weekends, were positively related to efficiency, and the number of staff contributed to a greater extent than the number of opening days. One possible explanation is that regardless of how many days are opened a week, the number of staff per POV per day is fixed and limited, and the maximum number of staff determines the maximum workload of a POV, which means that the efficiency may be more sensitive to staff numbers. This is consistent with the findings from prior studies about hospital efficiency [20].

As for the technical inputs, the proportion of staff with higher qualifications contributed positively to the efficiency. This is likely because those with higher education are more technically proficient in vaccination operations. In addition, the availability of information management logos was also positively associated with efficiency, which may be because the use of modern information technology allows POVs to have convenient functions such as online vaccination appointments and electronic information management. This is consistent with a study of hospital operational efficiency in India [13].

As for the external influencing factors, the Tobit model indicated that efficiency can be influenced by the following social environment variables. GDP was positively associated with vaccination efficiency. One possible explanation may be that the higher GDP means residents have a higher standard of living and higher demand for health services, which in turn put more pressure to increase efficiency. Moreover, financial resource allocation is also positively correlated with vaccination efficiency, which may be due to the fact that more financial input means more money to purchase advanced equipment, leading to higher efficiency. This is consistent with studies exploring the efficiency of hospital management [21–23] and government spending on health [24].

Also, we found the number of people served was positively associated with efficiency. Possible explanations are economic scale and the presence of incremental returns to scale in vaccination services, which is consistent with the findings of a study from Turkey on hospital efficiency [19]. In addition, the total dependency ratio was negatively associated with vaccination efficiency, which may be due to the fact that children and the elderly are the main vaccination population, and a high dependency ratio can place an excessive burden on POVs operations and ultimately bring about inefficiencies. This conclusion is consistent with the findings of studies on the factors influencing the technical efficiency of China’s medical and health institutions [15].

Although the regression coefficients of the annual means of regional PM2.5 is not significant, their negative correlation worths further discussion. Currently, studies have found that environmental pollution leads to an increase in health care expenditure for the residents [25] and health care utilization for specific diseases, such as respiratory diseases [26], eczema of the skin [27]. Therefore, we would propose a possible explanation that environmental pollution strengthens people's health awareness of environmental diseases and weakens their health awareness of vaccination, thus increasing the medical use of diseases caused by environmental pollution, such as respiratory diseases and skin diseases, decreasing the medical use of prevention, and creating a crowding-out effect of disease treatment on disease prevention, which leads to decrease in vaccination amount and decrease in the efficiency of vaccination.

### Limitations and strengths

There were some limitations in this study. Firstly, due to data availability, we included only five external social-environmental factors in the Tobit model, and future studies could consider more factors, such as regional fatality rates, regional life expectancy per capita, and regional birth rates. Secondly, although the association of individual input variables or individual external environmental variables with vaccine efficiency was explored, we did not investigate the path of improving the efficiency of vaccination service resource allocation from a configurational perspective, which can be further studied in future studies by using other methods such as fsQCA [15].

Despite the limitations, this work also has several strengths. To our knowledge, this is the first study to examine the influencing factors of vaccination service efficiency in China. The other available studies only calculated efficiency scores and conducted descriptive analysis [10–12], while we explored the extent to which input and socio-environmental factors affect the efficiency of vaccination services, which enriches the research on the factors influencing vaccination efficiency. In addition, previous research assessed the efficiency of childhood vaccination [10–12], our input–output element includes both childhood and adult vaccines, so our findings measure the total efficiency of childhood and adult vaccination, which can provide a more comprehensive reference for vaccination policies.

## Conclusion

In conclusion, we found that the efficiency of vaccination services varied considerably across POVs in Beijing. Labor, capital, technology, and output factors were positively associated with efficiency. In addition, vaccination efficiency was influenced by the social environment external to the POVs. The policy implication of this study is to improve the efficiency of vaccination by investing more resources in factors such as medical staff, the computerization of POVs, etc., which have a greater impact on efficiency, to facilitate vaccination. Besides, the allocation of vaccination resources should consider the social environment of different regions, and invest more resources in those underdeveloped, low financial allocation, and large populations regions.

## Data Availability

The POVs level datasets used during the study are available from the corresponding author upon reasonable request. The regional-level datasets analyzed during the current study are publicly available from Beijing Municipal Bureau Statistics http://tjj.beijing.gov.cn/.
